# miR-26a inhibits invasion and metastasis of nasopharyngeal cancer by targeting EZH2

**DOI:** 10.3892/ol.2013.1173

**Published:** 2013-02-04

**Authors:** LI YU, JUAN LU, BAO ZHANG, XIONG LIU, LU WANG, SI-YANG LI, XIAO-HONG PENG, XIA XU, WEN-DONG TIAN, XIANG-PING LI

**Affiliations:** 1 Department of Otolaryngology-Head and Neck Surgery, Nanfang Hospital, Southern Medical University, Guangzhou 510515, P.R. China;; 2 School of Public Health and Tropical Medicine, Southern Medical University, Guangzhou 510515, P.R. China

**Keywords:** nasopharyngeal carcinoma, miR-26a, enhancer of zeste homolog 2, metastasis

## Abstract

Nasopharyngeal carcinoma (NPC) is a highly invasive and metastatic type of cancer that is widely prevalent in Southern China. Studies have shown that several microRNAs (miRNAs) are implicated in NPC metastasis. Our previous studies have demonstrated that miRNA miR-26a inhibits cell growth and tumorigenesis of NPC through the repression of enhancer of zeste homolog 2 (EZH2). However, the role of miR-26a in NPC metastasis remains unknown. In this study, we showed that ectopic expression of miR-26a inhibited the migratory and invasive capacities of NPC cells *in vitro*. Additionally, we used a murine model to investigate the role of miR-26a in NPC metastasis and results showed that miR-26a overexpression suppresses the metastatic behavior of NPC cells *in vivo*. Furthermore, the data demonstrated that miR-26a decreased the expression levels of EZH2 *in vitro* and *in vivo*, suggesting that the antimetastatic effect of miR-26a in NPC was mediated by regulating EZH2. Therefore, these findings indicate that miR-26a functions as an antimetastatic miRNA in NPC and that its antimetastatic effects are mediated mainly by repressing EZH2 expression.

## Introduction

Nasopharyngeal carcinoma (NPC) is a highly malignant tumor which is prone to metastasis. Three major etiological factors of NPCs are Epstein-Barr virus (EBV) infection, genetic alterations and environmental factors (1). The incidence of NPC has remained high in endemic regions. Southern China was reported to have the highest prevalence of this cancer in the world (20–50 cases per 100,000 individuals) (2). Compared with other head and neck squamous cell carcinomas, NPC patients tend to present at a more advanced stage with a higher metastatic potential. Once metastasis occurs, prognosis is poor (3,4). Therefore, a better understanding of the molecular mechanisms of NPC metastasis is vital for improving the prognosis of patients with NPC.

MicroRNAs (miRNAs) are an evolutionarily conserved family of small (∼22 nucleotides) non-protein-coding RNAs that suppress gene expression at a post-transcriptional level. They are increasingly recognized as key regulators of gene expression in multiple cellular activities, including tumorigenesis and metastasis in various tumors (5). In NPC, several miRNAs have been reported to act as oncogenes or suppressor genes, including miR-10b, which was shown to promote the metastasis of NPC cells in our previous studies (6–8). However, the mechanisms of NPC metastasis are yet to be elucidated.

We have previously demonstrated that miR-26a markedly suppresses cell proliferation by directly targeting enhancer of zeste homolog 2 (EZH2) in NPC (9). Our study further provided evidence that EZH2 supported the invasive capacity of NPC cells by inducing epithelial-mesenchymal transition (data not shown). Furthermore, a recent study also suggested the critical role of EZH2 in the control of cell invasion and metastasis by decreasing the expression levels of E-cadherin (10). However, the effect of miR-26a on migration and invasion in NPC remains undefined. In the present study, we provide results which show for the first time that miR-26a inhibits cell migration and invasion by attenuating EZH2 expression in NPC.

## Materials and methods

### Cell culture and miRNA transfection

The human NPC cell lines 5-8F and CNE2 were cultured in RPMI-1640 medium (Hyclone, Logan, UT, USA) with 10% fetal bovine serum (FBS; Hyclone) and 1% penicillin/streptomycin. The HEK293T cell line was cultured in DMEM/high glucose medium (Hyclone) with 10% FBS and 1% penicillin/streptomycin. All cells were maintained at 37°C with an atmosphere of 5% CO_2_. The miR-26a mimic, inhibitor, nonspecific negative control and inhibitor negative control were purchased from GenePhama (Shanghai, China) and transfected at a final concentration from 50 to 200 nM with Lipofectamine 2000 (Invitrogen, Carlsbad, CA, USA).

### Lentivirus production and stable transfection

Lentiviral vector production for miR-26a overexpression was carried out as described previously (9). An empty lentiviral vector was used as a control. The production, purification and titration of lentiviruses were performed as described previously (9). The packaged lentiviruses were named LV-miR-26a and LV-con. The NPC cell line 5-8F was infected with LV-miR-26a or LV-con to generate two stable cell lines: 5-8F/miR-26a with miR-26a overexpression and 5-8F/control as a control.

### Extraction of total RNA and quantitative real-time PCR (qPCR)

Total RNA was obtained from cells using RNAiso Plus (Takara, Shiga, Japan) and total RNA was reverse transcribed to cDNA using an All-in-One miRNA First-Strand cDNA Synthesis kit (GeneCopoeia Inc., Rockville, MD, USA) for miR-26a quantitation and a PrimeScript™ RT reagent kit (Takara) for EZH2 mRNA quantitation. qPCR was performed using All-in-One™ qPCR Mix (GeneCopoeia Inc.) on an ABI 7500HT System (Applied Biosystems, Carlsbad, CA, USA). U6 small nuclear RNA (U6) or GAPDH was used as the endogenous control. Gene expression was normalized to internal controls and fold changes were calculated using relative quantification (2^–ΔΔCt^) (11).

### Western blot analysis

Protein lysates were separated by SDS-PAGE and electrophoretically transferred to polyvinylidene difluoride (PVDF) membranes (Millipore, Billerica, MA, USA). The membrane was incubated with a rabbit monoclonal antibody against human EZH2 (1:500 dilution, Cell Signaling Technology, Inc., Danvers, MA, USA) followed by HRP-labeled goat anti-mouse IgG (Santa Cruz Biotechnology, Inc., Santa Cruz, CA, USA) and detected by chemiluminescence. ACTB was used as a protein loading control.

### In vitro migration and invasion assays

For transwell migration assays, 5×10^4^ cells were plated in the top chamber onto the non-coated membrane (24-well insert; pore size, 8 μm; Corning, NY, USA). For invasion assays, 1×10^5^ cells were plated in the top chamber onto the Matrigel-coated membrane (24-well insert; pore size, 8 μm; BD Biosciences, Franklin Lakes, NJ, USA). The cells were incubated for 24 h and cells that did not migrate or invade through the pores were removed using a cotton swab. Cells on the lower surface of the membrane were fixed with 4% paraformaldehyde, stained with 0.5% crystal violet and then counted. Each experiment was performed in triplicate and the data are expressed as mean ± standard error of the mean (SEM) from 3 independent experiments.

### Mice xenografts

A total of 10 female BALB/c nu/nu nude mice (4 weeks old) were purchased from the Laboratory Animal Center of Southern Medical University (Guangzhou, China). The animal procedures were approved by the Animal Investigation Committee of Southern Medical University. The mice were anesthetized with 1% pentobarbital sodium (40 mg/kg) prior to surgery. Primary tumors were established by direct injection of 2×10^5^ cells into the liver as indicated in previously described methods (6). All mice were randomized into two groups (5–8F/control and 5–8F/miR-26a) and each group contained 5 mice. After 32 days, the mice were subjected to GFP fluorescence imaging and sacrificed and their livers and lungs were dissected out to perform GFP fluorescence imaging and pathological examination. Metastases in the liver and lungs were observed and counted.

### Histology and immunohistochemistry (IHC)

Paraffin-embedded tumor tissues were sectioned (4–6 μm) and used for histology and IHC analysis. IHC for EZH2 was performed using a Dako Envision System (Dako, Carpinteria, CA, USA) according to the manufacturer's instructions. The expression of EZH2 was examined in 5 randomly selected fields according to semiquantitative scales. The staining intensity was scored on a scale of 0 to 3, where 0 was negative, 1 was weak*,* 2 was medium and 3 was strong. The extent of the staining, defined as the percentage of positively stained cells relative to the whole field, was scored on a scale of 0 to 4, where 0 was 10%, 1 was 10–25%, 2 was 26–50%, 3 was 51–75% and 4 was ≥76%. The intensity score (0–3) was multiplied by the staining extent score (0–4), resulting in the final staining score for EZH2. For statistical analysis, a final staining score of 0–1, 2–4, 5–8 and 9–12 were considered to indicate negative, low, medium and high expression levels, respectively.

### Statistical analysis

SPSS 13.0 was used for statistical analysis. Data are presented as mean ± SEM of ≥3 independent experiments. A two-tailed Student's t-test was used for the comparison of two independent groups. Quantification of EZH2 by IHC was compared using a Mann-Whitney U test. P<0.05 was considered to indicate a statistically significant difference.

## Results

### miR-26a decreases the migratory and invasive capacity of NPC cells

**Figure 1:**
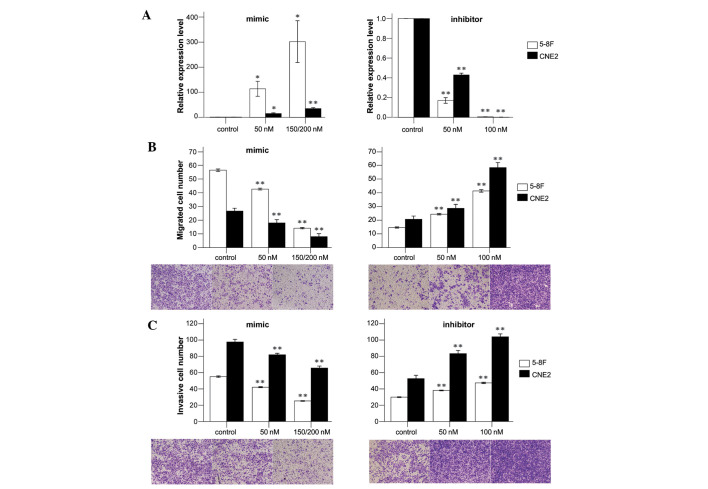
miR-26a inhibits 5-8F and CNE2 cell migration and invasion. (A) The expression levels of miR-26a in 5-8F and CNE2 cells, transfected with mimics or inhibitors at different concentrations. ^*^P<0.05, ^**^P<0.01 compared with the control. (B and C) The migrated or invasive cell numbers of 5-8F or CNE2 cells transfected with verified mimics or inhibitors. n=20, ^**^P<0.01 compared with the control. Representive fields of migrated or invasive cells on the membrane (magnification, x100).

To determine the expression levels of miR-26a in human NPC cells in response to the miR-26a mimic, inhibitor and negative control, qPCR was performed. Our results indicated that these oligonucleotides regulated the expression levels of miR-26a dose-dependently (Fig. 1A). For 5-8F cells transfected with a mimic, the relative expression levels of miR-26a were significantly enhanced compared with the control. At the final concentration of 200 nM, the expression levels of miR-26a were increased 301-fold. The relative expression levels of miR-26a were decreased by 80–99% when transfected with an inhibitor. Similar results were demonstrated in CNE2 cells (Fig. 1A).

To investigate the effects of dysregulated miR-26a on cell invasion and migration, we conducted cell migration and invasion assays with 5-8F and CNE2 cells. We showed that upregulated expression of miR-26a significantly suppressed the migratory and invasive abilities of 5-8F cells. The numbers of migrated and invasive cells were decreased by 68 and 36%, respectively, when 5-8F cells were transfected with a mimic at a final concentration of 150 nM. The numbers of migrated and invasive cells were increased by ∼2-fold and 50%, respectively, when transfected with an inhibitor (Fig. 1B and C). Similar results were demonstrated in CNE2 cells (Fig. 1B and C). These results emphasize the vital role of miR-26a in NPC metastasis.

### miR-26a suppresses the metastatic behavior of NPC tumors in vivo

**Figure 2:**
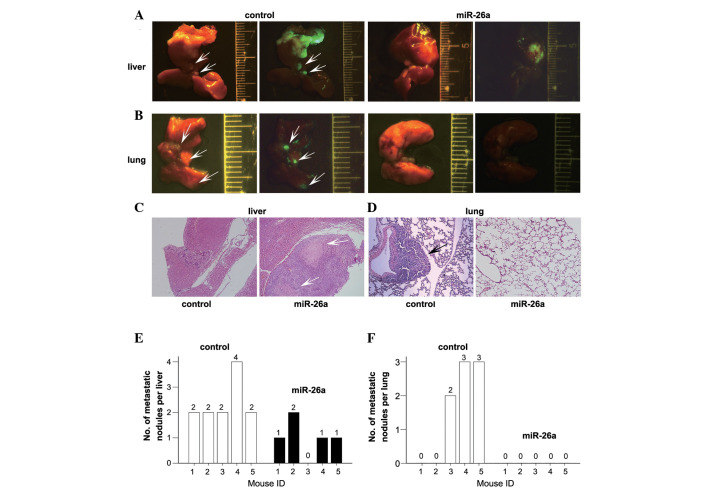
miR-26a suppressed the lung and liver metastases of NPC cells *in vivo*. Groups of BALB/c nude mice were inoculated with 5-8F/control or 5-8F/miR-26a cells. The liver and lung metastases were monitored by GFP-based fluorescence imaging on day 32 after inoculation (n=5 per group). (A) Livers from the mice of the control and miR-26a groups. White arrows indicate liver metastases. (B) Lungs of mice from the control and miR-26a groups. White arrows indicate lung metastases. The representative images of lung and liver tissues of two groups are presented. (C and D) Tissue sections were stained by H&E. (C) Necrosis in liver and (D) metastases in lung were detected (magnification, x100). White arrows, site of necrosis; black arrows, metastatic tumors. The representative images of lung and liver tissues of the two groups are presented. (E and F) Numbers of the metastatic nodules in the liver and lungs. NPC, nasopharyngeal carcinoma.

Lentiviral vectors were used to restore the expression of miR-26a in 5-8F and CNE2 cells in order to evaluate the effects of miR-26a overexpression in a murine model of NPC metastasis. The suppressive effects on cell migration and invasion induced by LV-miR-26a infection was similar to that induced by an miR-26a mimic transfection (data not shown). Primary tumors were established by direct injection of miR-26a-transduced or mock-infected 5-8F cells into the liver. The mice were sacrificed and autopsied on day 32 and the morphology of the liver and lungs was examined. As shown in Fig. 2A, the surface of the livers in LV-miR-26a-treated groups was smooth and had only a few metastatic tumors, with the exception of the region of the transplanted tumor. By contrast, the livers of the control group exhibited multiple metastatic tumors of various sizes on their surfaces. Additionally, the LV-miR-26a-treated mice showed normal lung morphologies with no indication of metastatic tumors, whereas the control group clearly exhibited multiple lung metastases (Fig. 2B). Consistent with the morphological observations, histological studies confirmed the presence of metastatic tumors in the lung tissue of LV-con-treated mice (Fig. 2D) and miR-26a overexpression induced large areas of necrosis in the primary tumor tissues (Fig. 2C). Compared with the control mice, none of the mice who had received heterotopic transplantation of miR-26a-overexpressing 5-8F cells exhibited lung metastases, however, 80% (4/5) of these mice developed liver metastases (Fig. 2E and F).

### miR-26a inhibits NPC metastasis by regulating EZH2

**Figure 3:**
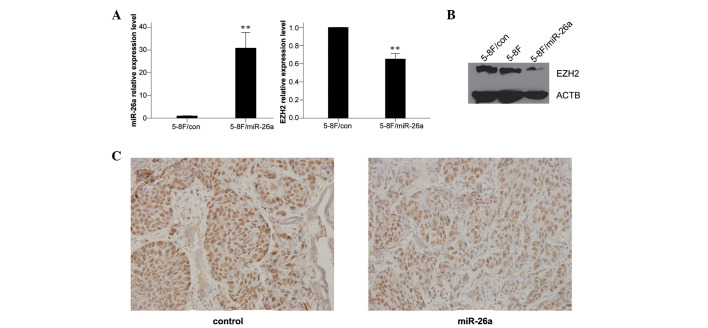
EZH2 was inversely correlated with miR-26a levels. (A) The expression levels of miR-26a and EZH2 in 5-8F cells transfected with LV-control and LV-miR-26a. ^**^P<0.01 compared with the control group. (B) The expression of EZH2 protein in cells transfected with LV-miR-26a was decreased compared with the control. (C) Immunohistochemistal staining of EZH2 in primary liver tumor tissues of NPC metastasis-bearing mice. The representative images are presented (magnification, ×100). EZH2, enhancer of zeste homolog 2; NPC, nasopharyngeal carcinoma.

EZH2 has been identified as the direct target of miR-26a in NPC and has been shown to repress E-cadherin expression to promote NPC metastasis (9,10,12). To explore the association between miR-26a and EZH2, we examined the expression levels of miR-26a and EZH2 in 5-8F cells transfected with LV-con or LV-miR-26a. The results indicated that miR-26a overexpression led to a decreased level of EZH2 mRNA and protein (Fig. 3A and B), which was consistent with our previous study (9). To further elucidate the role of EZH2 in the regulation of tumor metastasis by miR-26a, the primary tumor tissues of mice in each group were immunostained with an EZH2 antibody. The results showed that the expression levels of EZH2 were significantly reduced in the miR-26a-treated mice compared with the control group (Fig. 3C and Table I), indicating that miR-26a inhibited NPC metastasis by regulating EZH2.

## Discussion

Since the first miRNA was described in 1993, 1600 miRNAs have been identified in *Homo sapiens* according to miRBase (August 2012). Dysregulated expression of miRNA has been reported in numerous types of cancer and the majority of them function as tumor suppressors or oncogenes by regulating tumor cell proliferation, differentiation, apoptosis and metastasis. In this study, we explored the effects of miR-26a on metastasis in NPC.

We selected 5-8F and CNE2 from 7 NPC cell lines which presented with reduced expression levels of miR-26a (9) for *in vitro* experiments. The 5-8F cell line with high metastatic potential and the 6-10B cell line with no metastatic potential were generated from SUNE-1 cells. CNE2 was a poorly differentiated squamous cell line of NPC. Therefore, these two cell lines provided an excellent model for the investigation of the antimetastatic effects of miR-26a in NPC. A suitable model of NPC metastasis is likely to aid the elucidation of the mechanisms of metastasis and evaluate the potential for a novel treatment of metastasis. The orthotopic model closely simulated the clinical features of NPC growth (13), however, there appeared to be only a small chance of distant metastasis since the mice might succumb to complete airway occlusion caused by the exposed tumor. Previously, we have successfully established a murine model of NPC metastasis by inoculating C666-1 cells into the liver of a mouse as a single nodule, which subsequently metastasized to other parts of the liver and the lungs (14). In this study, using this metastatic model, we assessed the antimetastatic activities of miR-26a *in vivo*.

To investigate the role of miR-26a in NPC metastasis, we performed cell migration and invasion assays *in vitro* and examined the effects of miR-26a overexpression in a murine model of NPC metastasis. We showed that ectopic expression of miR-26a inhibited cell migration and invasion in a dose-dependent manner and the knockdown of miR-26a was able to create the opposite effect (Fig. 1). Consistent with the *in vitro* data, miR-26a overexpression significantly inhibited tumor growth and metastasis *in vivo* (Fig. 2). Thus, our data revealed miR-26a as a negative regulator of the metastasis in NPC. These results were consistent with findings in pancreatic cancer, in which miR-26a was downregulated and ectopic expression of miR-26a inhibited cell invasion and migration *in vitro*(15,16). However, a controversial role of miR-26a in lung cancer has been reported, which suggested miR-26a as a pro-metastatic miRNA (17). This suggests that the tissue- and time-dependent expression of miR-26a may affect its downstream targets to generate diverse funtions.

EZH2 is a member of the polycomb group of proteins which directly control DNA methylation (18). Numerous studies suggest that EZH2 is aberrantly overexpressed in several types of cancer, particularly metastasic breast and prostate cancer, and EZH2 overexpression promotes cell proliferation, invasion and metastasis (19–21). We have previously shown that EZH2 was a direct target gene of miR-26a in NPC (9). In this study, in accordance with former results, we showed that EZH2 mRNA and protein levels were attenuated by miR-26a. Moreover, the results of IHC demonstrated significantly lower EZH2 expression levels in primary tumors of the 5-8F/miR-26a group (Fig. 3), indicating EZH2 as the target gene of miR-26a in NPC metastasis. As shown in our previous studies, EZH2 alone was able to support the invasive capacity of NPC cells by inducing epithelial-mesenchymal transition (data not shown). This was further supported by results which demonstrated that EZH2 promoted NPC cell invasion by downregulating E-cadherin (10). In this study, we hypothesized that the antimetastatic effect of miR-26a in NPC was mediated through EZH2.

In conclusion, we have identified for the first time that miR-26a inhibits cell migration and invasion in NPC *in vitro* and *in vivo* and the inhibitory effects were at least partially mediated by EZH2. Since miR-26a is downregulated in NPC, re-introduction of this mature miRNA into the tumor tissue may provide a therapeutic strategy which reduces expression of the target genes. Although miRNA-based therapies remain in their infancy, our findings on miR-26a are encouraging and suggest that this specific miRNA may be a potential target for the treatment of NPC.

## Figures and Tables

**Table I:** Immunohistochemical detection of EZH2 in primary tumors in the control and miR-26a groups.

		EZH2				
Group	Fields	-	+	++	+++	P-value
Control	5	0	0	1	4	0.012
miR-26a	5	1	2	2	0	

EZH2, enhancer of zeste homolog 2.
